# Fluoxetine exposure alters behavior and morphology of *Xenopus laevis* tadpoles

**DOI:** 10.17912/micropub.biology.001505

**Published:** 2025-07-10

**Authors:** Luis J. Cordero, Zoe Smithwick, Berit Cederlund, Barbara Lom

**Affiliations:** 1 Neuroscience Program, Davidson College, Davidson, North Carolina, United States; 2 Neuroscience Program; Gender and Sexuality Studies Department, Davidson College, Davidson, North Carolina, United States; 3 Biology Department, Davidson College, Davidson, North Carolina, United States; 4 Biology Department; Neuroscience Program, Davidson College, Davidson, North Carolina, United States

## Abstract

This study exposed developing *Xenopus* tadpoles to the common antidepressant fluoxetine (FLU). Behaviorally, stage 46 control (0 nM) tadpoles significantly preferred white over black backgrounds, a potential anti-predation behavior, while tadpoles reared in 0.3 or 300 nM FLU did not display this preference. Anatomically, stage 46 tadpoles reared in 300 nM FLU appeared similar to controls (0 nM) yet measured slightly and significantly larger in six of seven aspects of gross morphology measured (total length, tail length, body length, body height, eye diameter, internasal distance). Consequently, developmental FLU exposure may subtly alter specific aspects of tadpole morphology and behavior.

**Figure 1. Developmental fluoxetine exposure alters behavior and morphology of Xenopus laevis tadpoles:**
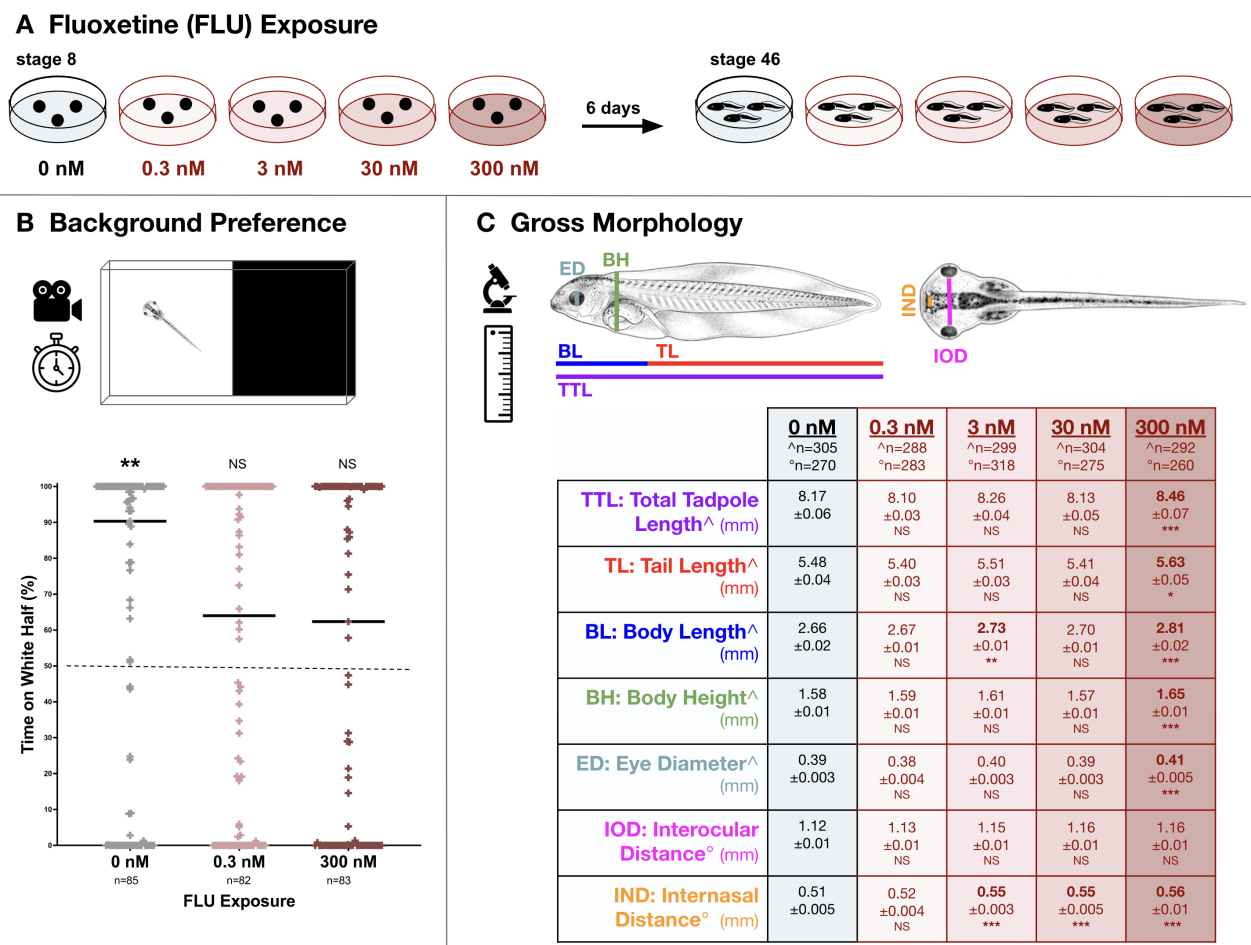
**A)** To determine the influence of the antidepressant fluoxetine (FLU) on early *Xenopus* *laevis* development embryos were reared in 0-300 nM FLU for six days to stage 46. **B)** Individual tadpoles were carefully transferred into the center of a half-white/half-black chamber to evaluate background preference. After a 30-second acclimation period tadpole position was then recorded for 120 seconds. Untreated (0 nM) stage 46 tadpoles spent significantly (p=0.0067) more of the observation period located in the white half (median=90%; horizontal line), a potential predation avoidance behavior. Tadpoles reared in 0.3 and 300 nM FLU did not exhibit a significant background preference, spending slightly though not significantly more time in the white half (medians=64% and 62% respectively; p>0.05). **C)** After chronic exposure to 0, 0.3, 3.0, 30, or 300 nM FLU seven anatomical features were measured to evaluate gross morphology in stage 46 tadpoles: total tadpole length (TTL), tail length (TL), body length (BL), body height (BH), eye diameter (ED), interocular distance (IOD), and internasal distance (IND). Interocular distance (IOD) was unaffected by all concentrations of FLU. Tadpoles reared in 300 nM FLU displayed small yet significant (p<0.05) increases in the remaining six morphological features (TTL, TL, BL, BH, ED, IND) compared to untreated controls (0 nM). Additionally, internasal distance (IND) was significantly larger for tadpoles reared in 3.0 and 30 nM FLU and body length (BL) was significantly longer for tadpoles reared in 3.0 nM. NS=not significant; * p<0.05, ** p<0.01; *** p<0.001. Tadpole illustrations in B and C © 2021 Natalya Zahn (www.xenbase.org RRID:SCR_003280; Zahn et al., 2017, 2022; CC BY-NC 4.0).

## Description

The selective serotonin reuptake inhibitor (SSRI) fluoxetine (FLU), also known as Prozac, is a commonly prescribed pharmacological treatment option for depression and related conditions (Kovich et al., 2023; Marx et al., 2023). FLU specifically enhances serotonin (5-HT) availability by slowing reuptake of this important neurotransmitter, stabilizing mood and reducing depressive symptoms (Bymaster et al., 2002; Pourhamzeh et al., 2022). Fluoxetine is generally considered safe to use during pregnancy (Kulin et al., 1998; Alwan et al., 2016; Ornoy and Koren, 2017; Bérard et al., 2019). Concerns regarding gestational FLU exposure, however, consider enhanced risk of preterm birth, reduced placental development, and low birth weight (Wen et al., 2006; Wisner et al., 2009; Domingues et al., 2023). Morphological alterations in cardiovascular and nervous systems as well as behavioral changes have also been observed in children exposed to FLU during gestation (Casper et al., 2003; Wen et al. 2006; Wogelius et al., 2006; Nijenhuis et al., 2011; Grigoriadis et al., 2013; Huybrechts et al., 2014; Kempny et al., 2014; Hanley et al., 2015; Reefhuis et al., 2015; Gao et al., 2017; van der Veere et al., 2020; Koc et al., 2023). FLU exposure during development is also associated with mild alterations of behavior, anatomy, and/or gene expression in vertebrate animal models (Vorhees et al., 1994; Rayen et al., 2011; Simpson et al., 2011; Barry, 2014; Hooper et al., 2016; Sprowles et al., 2017; Grieb and Ragan, 2019; Mikhailenko and Butkevich, 2019; Linhares et al., 2022; Vuong et al., 2021). Consequently, examining embryonic development in the presence of FLU may provide potential insights into relative benefits and risks of gestational SSRIs pharmacotherapy as well as the influences of serotonergic signaling during embryogenesis.

The *Xenopus laevis* embryo has long served as a powerful vertebrate model organism to study embryogenesis in part because the externally fertilized embryos are fully accessible for visualization throughout all stages of development and embryos are well suited for a wide variety of experimental manipulations (Pratt and Khakhalin, 2013; Hu et al., 2015; Sater and Moody, 2017; Exner and Willsey, 2021; Gao and Shen, 2021; Fainsod and Moody, 2022). Investigations of developmental effects of pharmacological and/or environmental agents are particularly amenable in *Xenopus* because soluble compounds of interest can be added to the solution in which the tadpoles are reared (Pratt and Khakhalin, 2013; Hu et al., 2015). Serotonin is expressed in early *Xenopus* embryos and plays key developmental roles well beyond facilitating synaptic transmission (Fukumoto et al., 2005; Nikishin et al., 2012; Berg et al., 2013). Interfering with serotonin during specific developmental periods of vertebrate development can lead to physical and neurological abnormalities (Reisoli et al., 2010; Beyer et al., 2012; Romero-Reyes et al., 2021). In tadpoles, FLU exposure causes developmental delays, skull and craniofacial malformations, and altered dendritic morphologies (Foster et al., 2010; Calibuso-Salazar and Ten Eyck, 2015; Liu et al., 2021). Moreover, FLU exposure affects tadpole and frog behaviors, including reduced startle habituation, reduced schooling, altered motility and locomotion, increased seizure susceptibility, and compromised predator avoidance responses (Barry, 2014; Coleman et al., 2019; Liu et al., 2021). More broadly, FLU has been linked to disruptions in locomotion and survival-related behaviors across multiple aquatic vertebrate species (Airhart et al., 2007; Saaristo et al., 2017; Sehonova et al., 2019; Correia et al., 2023, 2024).

This study reared *Xenopus laevis* embryos in 0-300 nM FLU concentrations ( Fig. 1A ) for six days to stage 46 to examine how embryonic exposure to the SSRI influenced background preference behavior ( Fig. 1B ) and gross morphology ( Fig. 1C ). When given a choice between white and black backgrounds, tadpoles typically spend significantly more time on white backgrounds, a visually mediated behavior that is thought to reduce predation risk in natural aquatic settings, where dark areas can be associated with predators (Moriya et al., 1996; Viczian and Zuber, 2014; Bruno et al., 2022; Adebogun et al., 2023). When stage 46 tadpoles were carefully placed in the center of a chamber with a half-white, half-black background, allowed a 30-second acclimation period, then observed for 120 seconds, the untreated control tadpoles (0 nM) spent significantly (p=0.0067) more time in the white half of the chamber (median=90%; Fig. 1B ). Interestingly, tadpoles reared in both low (0.3 nM) and high (300 nM) FLU concentrations did not show significant preference for the white background (medians=62% and 62% respectively p>0.05; Fig. 1B ). The absence of background preference in FLU-treated tadpoles suggests that early and chronic FLU exposure may alter an innate protective behavior and thereby increase predation risk. Chronic FLU exposure (up to 9.7 nM) compromised a predator avoidance behavior in *Bufo arabicus* tadpoles (Barry et al., 2014) while acute 1,000 nM FLU treatment resulted in increased preferences for light backgrounds in older (stage 48-49) *Xenopus* tadpoles (Bruno et al., 2022). In adult *Xenopus* frogs predator avoidance behaviors were unaltered by FLU injections (Coleman et al., 2019; Menon et al., 2023).

In addition to background preferences, many animals exhibit positive thigmotaxis, or edge-hugging behaviors that are thought to reflect signs of anxiety and/or predator avoidance when exploring novel environments (Carlson and Langkilde, 2013). In this study, regardless of FLU treatment, tadpoles spent roughly half of the observation period in the center versus periphery of the white/black chamber indicating that tadpoles were motile. Developmental FLU exposures of 0.3 and 300 nM did not significantly alter thigmotaxis behavior in stage 46 *Xenopus* tadpoles (0 nM: 47.7 + 8.4% of time in the periphery (n=85) versus 49.7 + 8.6% for 0.3 nM (n=82) and 52.9 + 8.7% for 300 nM (n=83); p>0.05), similar to unaffected thigmotaxis behaviors observed in zebrafish larvae exposed to 6,470 nM FLU (Richendrfer et al., 2012).

To determine if fluoxetine (FLU) influenced *Xenopus laevis* visual discrimination of the white versus black backgrounds a non-behavioral visual response assay was adapted (Bertolesi et al., 2015, 2017, 2021). *Xenopus* tadpoles reared on solid white or black backgrounds typically adjust their pigmentation by constricting or expanding melanophores, a camouflage response that requires visual input from the eyes (Bertolesi et al., 2017). Pigmentation over the dorsal brain was measured in tadpoles raised on solid white or solid black backgrounds. Similar to prior observations (Bertolesi et al., 2015, 2017), control (0 nM) tadpoles displayed significantly (p<0.001) darker pigmentation when raised for six days on solid black backgrounds (22.4 + 1.5% pigmentation index; n=84) compared to siblings reared on solid white backgrounds (9.0 + 0.54% pigmentation index; n=71). Compared to untreated controls, 300 nM FLU exposure did not significantly (p=0.99) alter pigmentation values for tadpoles reared on black (21.7 + 1.2% pigmentation index; n=47) or white (8.1 + 0.25% pigmentation index; n=66) backgrounds. Consequently, it is unlikely that the lack of preference for the white background observed in tadpoles reared in FLU ( Fig. 1B ) was due to a reduction in sensory ability to discriminate between white versus black backgrounds.

Morphology was assessed at stage 46 to determine if FLU exposure during early development affected tadpole development. Tadpole gross anatomy appeared visually similar in all conditions (0-300 nM). Consistently low rates of embryos with atypical appearances were observed in all conditions (0 nM: 1.7% atypical; 0.3 nM: 1.0% atypical; 3 nM: 2.7% atypical; 30 nM: 2.4% atypical; 300 nM: 2.4% atypical) that did not differ significantly (p>0.05). In addition, seven gross anatomical features were measured ( Fig. 1C ). Tadpoles exposed to the lowest FLU concentration (0.3 nM) exhibited no significant changes in any of the seven morphological features compared to untreated controls. The interocular distance (IOD) was unaffected in all conditions (0-300 nM). In contrast, the remaining six morphological features of tadpoles reared in 300 nM FLU were slightly (~3-10%) yet significantly (p<0.05) enlarged compared to untreated controls: total tadpole length (TTL), tail length (TL), body length (BL), body height (BH), eye diameter (ED), internasal distance (IND). Additionally, at intermediate FLU doses, internasal distance (IND) was significantly larger for tadpoles reared in 3.0 and 30 nM FLU while body length (BL) was significantly larger for tadpoles reared in 3.0 nM FLU. These anatomical observations suggest that developmental FLU exposure can increase tadpole size, particularly at 300 nM. Prior studies observed that FLU delayed development and reduced post-metamorphosis weight in three frog species (Conners et al., 2009; Foster et al., 2010; Calibuso-Salazar and Ten Eyck, 2015). These post-metamorphosis size reductions contrast the slight increases in pre-metamorphosis measurements observed in this study of *Xenopus* tadpole development.

Taken together, the behavioral and anatomical results in this preliminary study offer insights into fluoxetine’s influences on early vertebrate development. Subtle changes in tadpole morphology and behavior observed here emphasize the importance of carefully understanding how exposure to a common antidepressant can influence aquatic vertebrate embryo development.

## Methods

All procedures were approved by the Davidson College Institutional Animal Care and Use Committee (IACUC). Adult *Xenopus laevis* were maintained in a dedicated vivarium space, housed as two to three female frogs and up to five male frogs per polycarbonate container (Sive et al., 2000). Environmental conditions were controlled, with temperatures maintained between 20-23 °C, with a light-dark cycle of 12:12 hours. To prepare for mating, female frogs were primed with an injection of 50 U of human chorionic gonadotropin (hCG) into the dorsal lymph sac approximately one week prior. To induce hyperovulation, females received an additional 350 U of hCG and males received 50 U of hCG then they were placed together overnight in a container. The following morning, embryos were retrieved and dejellied in 2% cysteine in 20% Steinberg’s solution at pH 7.5 (recipe per Lom and Cohen-Cory, 1999) for 1-2 minutes, then thoroughly rinsed with at least three changes of 20% Steinberg's solution. Embryos with visibly atypical cleavage patterns were discarded.

A stock solution of 1 mg/L FLU in 20% Steinberg’s solution was stored at 4 °C and diluted to concentrations of 0.3, 3, 30, and 300 nM immediately before use similar to Foster et al. (2010). Groups of fifty cleaving, stage 8 embryos were reared in 100 x 25 mm disposable Petri dishes containing 50 mL of 0, 0.3, 3, 30, or 300 nM FLU at 20 °C with ambient light ( Fig. 1A ). Solutions were refreshed daily until six days post fertilization when the tadpoles had developed to stage 46.

For behavioral testing, a black-white background assay (Viczian and Zuber, 2014) was adapted by using a transparent plastic chamber (12.4 cm x 8.4 cm x 3.0 cm) that had been painted half black and half white on its exterior and filled with ~75 mL of 20% Steinberg’s solution ( Fig. 1B ). After six days of development in 0, 0.3, and 300 nM FLU, a subset of stage 46 tadpoles were randomly selected for behavioral testing during the daytime when light preferences are strongest (Bruno et al., 2022). In a closed room with consistent lighting and a single experimenter, each tadpole was carefully placed into the center of the black/white chamber. After a 30-second acclimation period during which the tadpole explored the chamber, behavior was recorded for the subsequent 120 seconds using an iPhone 12 Pro Max camera at 4.5X zoom. After every trial the chamber was rotated before the next tadpole was tested. Recordings were converted from MOV to MP4 files with CloudConvert or FreeConvert for subsequent analysis with EthoVision XT 16 software to calculate the total time spent in the black versus white halves of the chamber as well as the total time in the central versus peripheral (outer 3.67 cm) regions of the chamber (thigmotaxis) using center-point detection, counter-based conformity, and activity analysis and adjusted for static detection. Statistical significance was assessed via one-way ANOVA using Prism software.

To determine if early exposure to FLU affected *Xenopus* visual function via a measurement independent of behavior a melanophore pigmentation assay was adapted (Bertolesi et al., 2015, 2017, 2021). Embryos were reared in 0 or 300 nM FLU for six days inside an environmental chamber set to a 12:12-hour light-dark cycle at 20 °C with the addition of solid black or white paper attached to the underside of the clear plastic dishes. After six days of FLU exposure stage 46 tadpoles were anesthetized with 0.5% tricaine and then fixed in 4% paraformaldehyde with 4% sucrose for at least four hours, followed by three washes in phosphate-buffered saline (PBS). Fixed tadpoles were positioned on 2% agarose cushions and their dorsal anterior pigmentation was imaged using a stereomicroscope with consistent lighting and magnification conditions. Using ImageJ, an experimenter blind to the treatment condition of each image drew a 500 x 200-pixel rectangle over the dorsal brain and then consistently thresholded the images so the percent area covered by melanophores (pigmentation index) could be determined. Statistical significance was assessed via one-way ANOVA using Prism software.

To determine if FLU influenced gross morphology, tadpoles were reared in 0-300 nM FLU for six days, anesthetized with 0.5% tricaine, then fixed in 4% paraformaldehyde with 4% sucrose for at least four hours, followed by three washes in phosphate-buffered saline (PBS). Tadpoles were positioned on a grooved 2% agarose cushion and imaged dorsally and laterally using a stereomicroscope. Seven gross morphological features were measured ( Fig. 1C ): total tadpole length (TTL), tail length (TL), body length (BL), body height (BH), and eye diameter (ED) were measured from lateral views and inter-nasal distance (IND), and interocular distance (IOD) were measured from dorsal views. Images were renamed using Advanced Rename software so that experimenters could manually measure lateral and dorsal features with ImageJ while unaware of treatment conditions. Lateral tadpole morphologies were categorized as typical or atypical per normal tables of *Xenopus* development (Nieuwkoop and Faber, 1994; Zahn et al., 2017, 2022). Statistical significance was determined via two-way ANOVA.

## Reagents

**Table:** 

**Reagent/Instrument**	**Source**	**Catalog #/Identifier**
agarose	Fisher Scientific	BP165
environmental chamber	Percival	n/a
fluoxetine hydrochloride	Sigma-Aldrich	F132
human chorionic gonadotropin (hCG)	Sigma-Aldrich	CG10
human chorionic gonadotropin (hCG)	Intervet	Chorulon
incubator	Revolutionary Science	Incufridge RS-IF-203
L-cysteine	Sigma Aldrich	C7880
paraformaldehyde	Sigma Aldrich	P6148
petri dishes (100 x 25 mm)	Fisherbrand	FB0875711
software - behavior analysis	Noldus	EthoVision XT 16
software - file conversion	Lunaweb	CloudConvert
software - file conversion	TRMedia	FreeConvert
software - graphing and statistics	GraphPad	Prism 10.4.1
software - image analysis	n/a (public domain)	ImageJ
software - image renaming	Hulubulu Software	Advanced Renamer
stereomicroscope	Nikon	SMZ1270
stereomicroscope	Meiji	EMZ-8TR
sucrose	Alpha Aesar	A15583
tricaine methanesulfonate	Western Chemical	Tricaine-S
video recorder	Apple	iPhone 12 Pro Max
